# The Molecular Structure of Human Red Blood Cell Membranes from Highly Oriented, Solid Supported Multi-Lamellar Membranes

**DOI:** 10.1038/srep39661

**Published:** 2017-01-03

**Authors:** Sebastian Himbert, Richard J. Alsop, Markus Rose, Laura Hertz, Alexander Dhaliwal, Jose M. Moran-Mirabal, Chris P. Verschoor, Dawn M. E. Bowdish, Lars Kaestner, Christian Wagner, Maikel C. Rheinstädter

**Affiliations:** 1 Department of Physics and Astronomy, McMaster University, Hamilton, ON, Canada; 2 Department of Experimental Physics, Saarland University, Saarbrücken, Germany; 3 Research Center for Molecular Imaging and Screening, Saarland University, Homburg/Saar, Germany; 4 Department of Chemistry and Chemical Biology, McMaster University, Hamilton, ON, Canada; 5 Department of Pathology and Molecular Medicine, McMaster University, Hamilton, Canada; 6 McMaster Immunology Research Center, McMaster University, Hamilton, Canada; 7 Michael G. DeGroote Institute for Infectious Disease Research, McMaster University, Hamilton, Canada

## Abstract

We prepared highly oriented, multi-lamellar stacks of human red blood cell (RBC) membranes applied on silicon wafers. RBC ghosts were prepared by hemolysis and applied onto functionalized silicon chips and annealed into multi-lamellar RBC membranes. High resolution X-ray diffraction was used to determine the molecular structure of the stacked membranes. We present direct experimental evidence that these RBC membranes consist of nanometer sized domains of integral coiled-coil peptides, as well as liquid ordered (*l*_*o*_) and liquid disordered (*l*_*d*_) lipids. Lamellar spacings, membrane and hydration water layer thicknesses, areas per lipid tail and domain sizes were determined. The common drug aspirin was added to the RBC membranes and found to interact with RBC membranes and preferably partition in the head group region of the *l*_*o*_ domain leading to a fluidification of the membranes, *i.e.*, a thinning of the bilayers and an increase in lipid tail spacing. Our results further support current models of RBC membranes as patchy structures and provide unprecedented structural details of the molecular organization in the different domains.

The presence of pale cells with no internal content in a blood smear is typically indicative of a disease. These cells are produced by hemolysis and have been named red blood cell (RBC) ghosts based on their appearance under the microscope. RBC ghosts can be prepared artificially^1^,^2^,^3^. The first published protocol in 1963 by Dodge, Mitchell and Hanahan describes the extraction of the cell membrane from RBCs through hemolysis and was an essential step in the development of membrane proteomics and lipidomics^4^,^5^.

While there is detailed knowledge of the composition of RBC membranes, information about the molecular organization of these components in the actual membranes is scarce^6^. This is, in particular, a consequence of the lack of suitable experimental techniques. The disordered, patchy and highly dynamic state of biological materials impedes, for instance, the use of high-resolution diffraction techniques as in protein crystallography. To overcome this constraint, we prepared highly oriented stacks of RBC membranes on silicon wafers and studied their molecular properties *in-vitro* using high resolution X-ray diffraction. The results present evidence for nanometer-sized domains of coiled-coils peptides, as well as liquid ordered (*l*_*o*_) and liquid disordered (*l*_*d*_) lipid patches, and give a detailed picture of the molecular organization in these domains.

When present in the body, aspirin (acetylsalicylic acid, ASA) and its metabolites interact with the cyclo-oxygenase (COX) pathway. The inhibition of both COX isoforms, COX-1 and COX-2, by higher dose aspirin is believed to lead to analgesic and anti-inflammatory effects, while lower doses, sufficient to inhibit COX-1 activity, lead to anti-platelet activity^7^,^8^. There is recent evidence that membrane composition and fluidity play an important role in platelet cell function^9^,^10^,^11^, possibly related to the formation of rafts^12^.

We present direct experimental evidence that aspirin incorporates into the head group region of erythrocytic membranes and leads to an increase in lipid tail distances and a decrease in membrane width, indicating increased membrane fluidity. ASA was found to preferably interact with the head group region of *l*_*o*_ domains of the RBC membranes.

## Results

**Figure 1:**
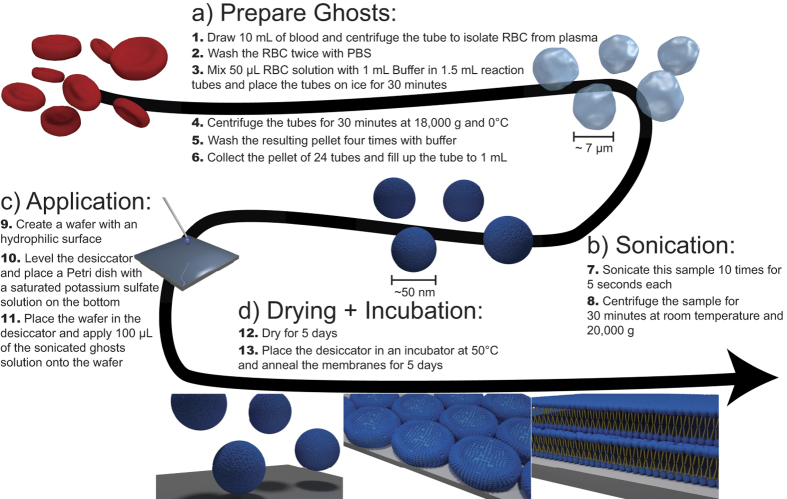
Schematic of the Blood-on-a-chip preparation protocol. The protocol is based on the original protocol for the preparation of red blood cell ghosts (**a**). The RBCs are then sonicated to form small vesicles with a uniform size distribution and centrifuged (**b**) before the solution is applied to silicon wafers (**c**). The membranes are dried and annealed (**d**) to form well developed multi-lamellar stacks of red blood cell membranes supported on silicon wafers.

The preparation protocol is schematically depicted in Fig. 1 and consists of two main parts: In the first step, RBC ghosts are produced from blood samples. In the second step, these RBC ghosts are applied onto silicon wafers and annealed to form multi-lamellar RBC membrane stacks. The detailed protocols are presented in the Materials and Methods Section.

### Molecular structure and properties of RBC membranes

**Figure 2:**
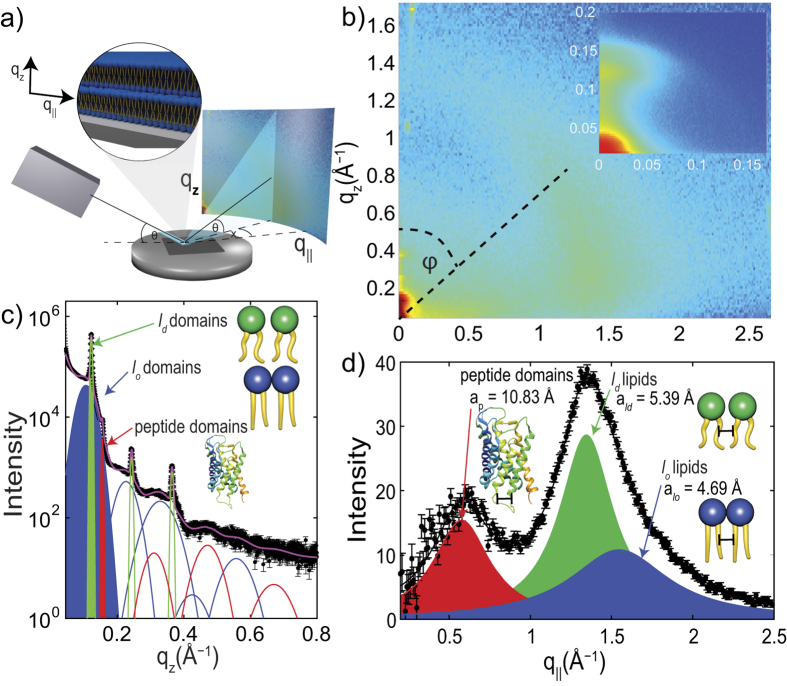
Overview of the X-ray diffraction results. The setup is schematically shown in (**a**). The highly aligned membranes are oriented on the X-ray diffractometer, such that *q*_*z*_ measures out-of-plane, and *q*_||_ in-plane membrane structure. (**b**) Two-dimensional data. The main features are a series of intensities along the *q*_*z*_-axis and three broad signals along the in-plane axis *q*_||_. (**c**) Shows a cut along *q*_*z*_. The data are well fit by three series of Bragg peaks corresponding to three different lamellar spacings assigned to *l*_*o*_ and *l*_*d*_ lipid domains (green and blue) and coiled-coil *α*-helical peptide domains (red). (**d**) The in-plane signals show three correlation peaks corresponding to the packing of *α*-helices in the peptide domains (*a*_*p*_ = 10.83 Å), and packing distances of *l*_*d*_ (*a*_*ld*_ = 5.39 Å) and *l*_*o*_ lipid tails (*a*_*lo*_ = 4.69 Å) in the hydrophobic membrane core.

X-ray diffraction was used to determine the molecular structure of the RBC membranes. As the membranes were oriented with their membrane plane parallel to the silicon substrate, the in-plane and out-of-plane structure was determined separately, but simultaneously. A schematic of the X-ray diffraction setup is shown in Fig. 2(a). Figure 2(b) shows typical 2-dimensional X-ray diffraction data. The organization of the membranes normal to the silicon wafer is observed along the *q*_*z*_-axis, while molecular organization in the plane of the membranes, parallel to the substrate, is observed along the *q*_||_-direction. Cuts of the diffracted intensity along the out-of-plane and in-plane direction are shown in Fig. 2(c) and (d). By analyzing out-of-plane and in-plane patterns, the corresponding signals were assigned to three different structures: liquid disordered (*l*_*o*_) and liquid disordred (*l*_*d*_) lipid domains, as well as membrane embedded peptides, as will be detailed in the next section.

#### Structure perpendicular to the membrane plane

**Figure 3:**
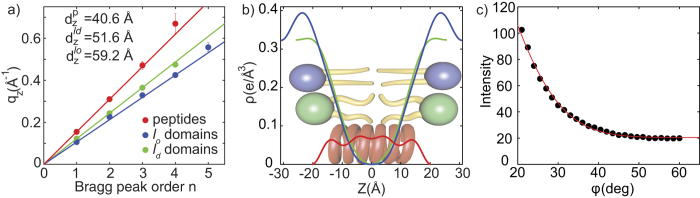
Analysis of the X-ray diffraction data in Fig. 2. The lamellar spacings of the peptide, as well as the *l*_*o*_ and *l*_*d*_ lipid domains are determined from the slopes of *q*_*z*_ *vs. n* plots. (**b**) Shows the corresponding electron densities, as determined through Fourier analysis of the out-of-plane diffraction data. The densities for the *l*_*o*_ and *l*_*d*_ lipid domains agree well with densities reported in the literature. The peptide domain shows an almost constant density in the hydrophobic membrane core, indicative of trans-membrane peptides. (**c**) Membrane orientation is determined from radial integration of the scattered intensity along the meridional degree, *φ*. The solid line is a fit using a Gaussian profile. By using Hermans orientation function, RBC membranes are 90.9% oriented with respect to the silicon substrate.

A lamellar membrane structure, *i.e.*, a stack of membranes, where the bilayers are organized parallel to each other, results in a series of equally spaced and well defined Bragg reflections in diffraction experiments^13^, corresponding to the ‘fundamental’ and the ‘overtones’. The well developed Bragg peaks along the out-of-plane axis in Fig. 2(c) indicate a lamellar organization of the RBC membranes on the substrate. The fundamental reflection for each series is colored in the Figure. Following Bragg’s law (*q*_*z*_ = 2*π*/*d*_*z*_ ⋅ *n*), the lamellar spacing, *d*_*z*_, can be determined from the slope of the curve when plotting *q*_*z*_ *vs.* the order of the Bragg peak, *n*. This is shown in Fig. 3(a). Three *d*_*z*_-spacings were determined: 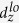 = 59.2 Å, 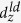 = 51.6 Å and 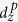 = 40.6 Å.

Electron density profiles, *ρ*(*z*), of the bilayers were determined through Fourier analysis of the out-of-plane Bragg peaks, as described in the Materials and Methods Section, and are shown in Fig. 3(b). The electron rich head group can be identified by the absolute maximum in the electron density profile at |*z*| ~ 22 Å. *ρ* monotonically decreases towards the bilayer center at *z* = 0, where CH_3_ groups typically reside, with a low electron density. The electron density profile of the 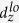 = 59.2 Å domain (blue curve) agrees well with a lipid bilayer with lipids in a well ordered, gel-like state with their lipid chains in an all-trans configuration^14^,^15^. Together with the information from the in-plane diffraction below, these signals were assigned to lipids in *l*_*o*_ domains. These domains are likely enriched in cholesterol, making them more ordered and thicker^16^,^17^.

The electron density corresponding to the 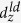 = 51.6 Å spacing (green curve) agrees well with the electron density reported for fluid lipid bilayers, where the structure of the lipid tails in the hydrophobic membrane core is dominated by gauche-defects, as reported for instance by^18^,^19^. Taking into account the in-plane diffraction analysis, these signals were assigned to domains of *l*_*d*_ lipids.

The 3^*rd*^ lamellar spacing of 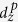 = 40.6 Å is significantly smaller than the spacings above, and the electron density is almost constant in the hydrophobic membrane core. This density profile is well described by *α*-helical coiled-coil peptides, which are embedded in the membranes^20^, as will be discussed below, and was assigned to domains of integral proteins.

Lamellar spacings, *d*_*z*_, membrane thicknesses, *d*_*HH*_, and the thicknesses of the water layer, *d*_*w*_ were determined from the electron densities and are listed in Table 1.

#### In-plane Membrane Structure

Three peaks, at *q*_||_ = 0.58 Å^−1^, *q*_||_ = 1.35 Å^−1^ and *q*_||_ = 1.55 Å^−1^, were observed in the in-plane diffraction in Fig. 2(d). These peaks fit well to distances between peptides and lipids, as observed in previous investigations in single and multi-component artificial and biological membranes^17^,^18^,^20^,^21^,^22^,^23^. The lipid in-plane peaks are the result of a hexagonal packing of the lipid tails in the hydrophobic membrane core (planar group p6)^16^. The distance between two acyl tails is determined using 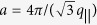, where *q*_||_ is the position of the corresponding correlation peak. The area per lipid chain is obtained to 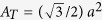. We note that this area also includes the area of cholesterol molecules.

It was reported that RBC lipid bilayers consist of equal amounts of cholesterol and phospholipids, such as phosphatidylcholine, sphingomyelin, phosphatidylethanolamine and phosphatidylserine^6^. The peaks at *q*_||_ = 1.35 Å^−1^ and *q*_||_ = 1.55 Å^−1^ are in good agreement with structural features reported in model lipid membranes in their well ordered gel and fluid phases, where the lipids tails take an all-trans conformation (gel) or are dominated by gauche defects (fluid). A correlation peak at ~1.5 Å^−1^ was reported in the gel phase of saturated phospholipid membranes, such as DMPC (Dimyristoyl-sn-glycero-3-phosphocholine) and DPPC (Dipalmitoyl-sn-glycero-3-phosphocholine)^14^,^16^,^21^,^24^. Unsaturated lipids were reported to order in a structure with slightly larger nearest neighbor tail distances, leading to an acyl-chain correlation peak at ~1.3 Å^−1^, as reported for DOPC and POPC^25^,^26^. These correlation peaks were, therefore, assigned to the *l*_*o*_ and *l*_*d*_ lipid components of the plasma membranes.

Lipid tail distances in *l*_*o*_ and *l*_*d*_ domains and lipid tail areas are listed in Table 1. Distances and areas in the *l*_*o*_ domains are smaller, as lipid tails in their all-trans configuration are straighter and pack tighter than *l*_*d*_ tails, dominated by gauche defects.

Membrane peptides are often organized in bundles, whose structure is dominated by *α*-helical coiled-coils^20^,^27^,^28^,^29^,^30^. Coiled coils consist of *α*-helices wind together to form a ropelike structure stabilized by hydrophobic interactions, and this structure is found in about 10% of the proteins in the human genome^31^. The main features of this motif is a ~10.8 Å (*q*_||_ ~ 0.58 Å^−1^) equatorial reflection corresponding to the spacing between adjacent coiled-coils^32^,^33^,^34^. This correlation peak is observed in the in-plane data in Fig. 2(d) (in red).

The volume fractions of the peptide, the *l*_*o*_ and *l*_*d*_ lipid domains were determined from the integrated peak intensities of the lipid and peptide signals in Fig. 2(d) to 30:45:25 (*l*_*o*_ lipids:*l*_*d*_ lipids:coiled peptides).

#### Membrane orientation

The orientation of the RBC membranes in the stack with respect to the silicon wafer was determined from the 2-dimensional data in Fig. 2(b) by radial integration using Hermans orientation function, as described in the Materials and Methods Section. The intensity of the first reflectivity peak as function of the meridional angle *φ* is plotted in Fig. 3(c), and the degree of orientation was determined to be 90.9% (±0.3%). While values of ~97% are reported for synthetic supported membranes (see, *e.g.* ref. 35), the value for RBC membranes is to the best of our knowledge the highest ever reported for a biological membrane. This high degree of orientation of the RBC membranes on silicon chips is required for a detailed structural characterization of the membranes, in particular to differentiate in-plane and out-of-plane structure.

#### Determination of domain size

The in-plane diffraction signals in Fig. 2(d) are significantly broader than typical Bragg peaks in crystalline materials, indicating that the corresponding domains are small. The domain sizes were estimated from the peak widths of the corresponding correlation peaks using Scherrer’s equation (as detailed in the Materials and Methods Section). Values for the domain size, *ξ*, are listed in Table 1. From these results, RBC membranes consist of small, nanometer sized domains of *l*_*o*_ and *l*_*d*_ lipids and coiled-coil *α*-helical peptides.

### The effect of aspirin on RBC membrane structure

**Figure 4:**
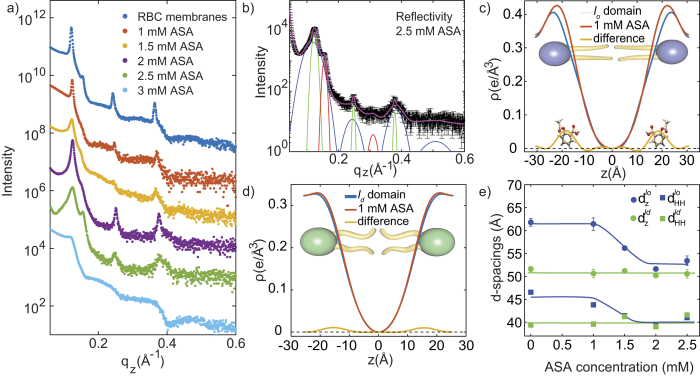
Analysis of the RBC/aspirin complexes. (**a**) Shows all reflectivity curves for complexes containing between 0 and 3 mM ASA. (**b**) The pattern for the 2.5 mM sample is well fit by three series of peaks corresponding to *l*_*o*_, *l*_*d*_ and peptide domains. (**c**) The location of the ASA molecule is determined by comparing the electron density of a pure RBC membrane with a low concentration of 1 mM ASA. Aspirin is found to partition the *l*_*o*_ lipid domains of RBC membranes and locate in the head group region, at |*z*|-values of 22.8 Å. (**d**) Small partitioning of aspirin is observed in *l*_*d*_ lipid domains, suggesting that aspirin preferably interacts with *l*_*o*_ domains. (**e**) Lamellar spacing, *d*_*z*_, and membrane thickness, *d*_*HH*_, of the *l*_*o*_ lipid domains decrease significantly with increasing ASA concentration until thickness of *l*_*o*_ and *l*_*d*_ domains coincide. We note that due to the absence of reflectivity peaks in the 3 mM ASA curve in part (a) no *d*-spacings could be determined for this concentration in part (e).

The out-of-plane scattering for RBC membranes containing 1 mM, 1.5 mM, 2 mM, 2.5 mM and 3 mM ASA is shown in Fig. 4(a). The curve containing 2.5 mM ASA and the corresponding fit is shown in part (b). Data is well fit by 3 series of Bragg peaks, corresponding to *l*_*o*_, *l*_*d*_ and peptide domains, in agreement with pure RBC membranes. Electron density profiles of the *l*_*o*_ lipid domain for RBC membranes and RBC membranes +1 mM aspirin are shown in Fig. 4(c). Upon the addition of aspirin, the electron density increases at *z* ~ 22.8 Å. Under the assumption that a small amount of aspirin does not disturb the bilayer structure significantly, the two densities can be subtracted and the extra intensity assigned to aspirin molecules. The experiments thus locate aspirin inside the head group region of the RBC membranes, in agreement with results in model phospholipid bilayers^15^,^36^,^37^. There is only a small effect of aspirin on the electron density of the *l*_*d*_ domains, as shown in Fig. 4(d), indicating that aspirin preferably interacts with *l*_*o*_ regions.

The lamellar spacing, *d*_*z*_, and head group to head group spacing, *d*_HH_, of the *l*_*o*_ and *l*_*d*_ lipid domains as function of ASA content are depicted in Fig. 4(e). While lamellar spacing and membrane thickness for the *l*_*d*_ lipid domains are not affected by the presence of ASA, the two spacings significantly decrease with increasing aspirin concentrations for the *l*_*o*_ lipid domains. They decrease until lamellar spacing and membrane thickness for *l*_*o*_ and *l*_*d*_ domains coincide at a ASA concentration of 2.5 mM. At this ASA concentration, the overall lamellar spacing of the RBC membranes is reduced to 53.4 Å; the overall membrane thickness to 41.8 Å.

Figure S2(a) in the Supplementary Material displays the *q*_||_ position of the *l*_*o*_ and *l*_*d*_ lipid in-plane peaks as function of aspirin concentration. While the lipid spacing in the *l*_*d*_ domain is unchanged by the presence of aspirin, the *q*_||_-value of the *l*_*o*_ signal slightly shifts to smaller *q*_||_-values, resulting in an increase in the distance between lipids from *a*_*lo*_ = 4.69 Å to *a*_*lo*_ = 4.85 Å. By using the relation 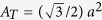, this increase results in an increase in tail area from 19.04 Å^2^ to 20.37 Å^2^.

From the analysis of the widths of the lipid and peptide correlation peaks, lipid domain sizes were found to be approximately independent of aspirin concentration (as shown in Fig. S2(b) in the Supplementary Material). However, a slight increase in peptide domain size was observed with increasing ASA content, from about 30 Å to about 38 Å.

## Discussion

In order to efficiently use biophysical techniques, such as fluorescence microscopy, atomic force microscopy, as well as X-ray and neutron scattering, highly oriented stacks of supported lipid bilayers are usually prepared^38^. These techniques are ideally suited to study molecular structure and dynamical properties of membranes^13^,^21^,^39^,^40^,^41^,^42^,^43^,^44^,^45^,^46^. The approach has advanced significantly during the past decades and is now used to study complex, multi-component membranes and their interaction with drugs, small molecules^13^,^20^,^26^,^37^,^47^,^48^,^49^,^50^,^51^,^52^,^53^, bacteria^54^,^55^, and in particular lipid rafts, *i.e.* functional lipid domains^16^,^17^,^56^,^57^,^58^,^59^,^60^. The preparation protocol presented in this paper produces highly oriented, multi-lamellar RBC membranes on silicon wafers, which are highly suited to provide detailed molecular level information. The combination of oriented membranes and state-of-the-art diffraction equipment and analysis gives unprecedented insight into the structure of these RBC membranes.

In early X-ray diffraction studies of human erythrocytes membranes^61^,^62^,^63^, ghosts were prepared using the Dodge protocol and pellets of the final preparation were imaged. Diffraction patterns with lamellar periodicities between ~55 and ~70 Å were observed and assigned to hemoglobin free membranes, in agreement with our findings. Large amounts of hemoglobin were reported to result in much larger lamellar periodicities of ~110 Å^61^. The electron density in Fig. 3b) agrees qualitatively with the early electron density in ref. 62, which was assigned to intact, hemoglobin-free erythrocyte membranes. However, the low purity and low degree of order in the RBC pellets likely prohibited a more detailed structural analysis at this time.

**Figure 5:**
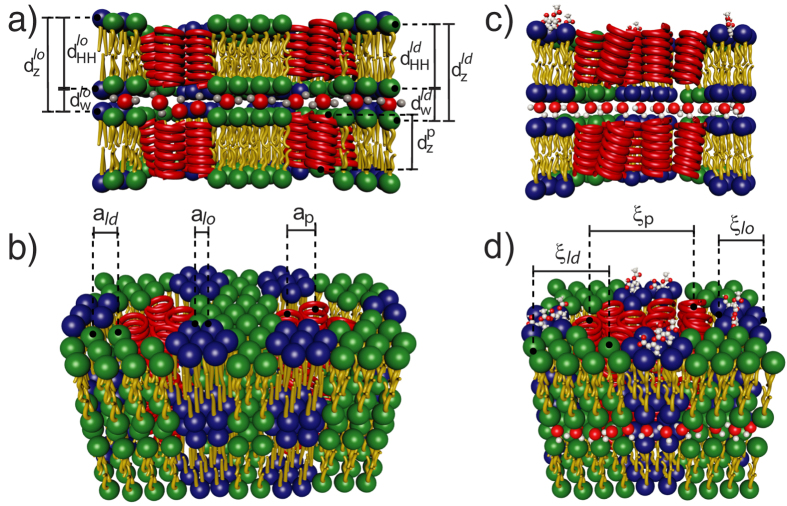
The structural findings are summarized in cartoons of pure RBC membranes in (**a**) and (**b**), and RBC membranes containing aspirin in (**c**) and (**d**). The images show side and top views. Structural parameters, such as the lamellar spacing, *d*_*z*_, the head group to head group thickness of the membranes, *d*_*HH*_, the thickness of the water layer, *d*_*w*_, and in-plane distances between lipid tails and integral peptides, *a*, and domain sizes, *ξ*, were determined for the *l*_*o*_ and *l*_*d*_ lipid, and the peptide domains. While the *l*_*o*_ and *l*_*d*_ lipid domains showed significantly different membrane thicknesses in pure RBC membranes, the addition of aspirin led to an overall thinning of the membranes and an increase of the lipid spacings, indicative of a fluidification. Aspirin was found to interact mainly with the *l*_*o*_ lipid domains.

From X-ray diffraction along the membrane normal, the lamellar spacing, *d*_*z*_, the membrane thickness, *d*_*HH*_, and the thickness of the water layer, *d*_*w*_, were determined and are listed in Table 1. From in-plane diffraction data, molecular distances and areas were determined. The out-of-plane and in-plane data in particular present evidence for a patchy structure: these RBC membranes consist of nanometer-sized liquid ordered (*l*_*o*_) and liquid disordered (*l*_*d*_) lipid domains as well as *α*-helical coiled-coil peptide domains (integral proteins), at ratios of 30.2% *l*_*o*_, 45.0% *l*_*d*_ and 24.8% coiled peptides. By assigning the scattering signals to the different phases, we could determine structural parameters for these domains separately. The structure of the multi-lamellar, solid supported RBC membranes is pictured in Fig. 5(a) and (b).

The structure of RBC membranes is no longer thought to be uniform, but to show ‘rafts’, regions enriched in cholesterol and sphingolipids in association with specific membrane proteins^6^. Rafts are typically thought to be a manifestation of the liquid ordered phase and, as such, enriched in cholesterol, thicker and with lipids showing gel-like properties. In nonerythroid cells, lipid rafts are suspected to be relevant for cell signaling events. In erythroid cells, they have been shown to mediate *β*2-adregenic receptor signaling and increase cAMP levels, and thus regulating entry of malarial parasites into normal red cells^64^. Properties and even existence of rafts are, however, a topic of intense debate in the literature^57^,^59^,^60^. The source of this debate is that we picture rafts as very small and highly dynamics structures, which are very difficult to observe. It is challenging for experimental techniques to cover the small length scales and fast dynamics at the same time.

Heterogeneities have indeed been observed in human erythrocytes. The main technique used is detergent-resistant-membranes (DRM), a membrane fraction resisting solubilization by a detergent^65^. Recently, raft-like heterogeneities have also been reported from fluourescent labeling techniques in live erythrocytes^65^,^66^. The structures observed in this paper are in very good agreement with the raft hypothesis: they are small, nanometer-sized patches and the lipids in one of the patches appear indeed to be more gel-like and the corresponding patches to be thicker. Those patches, therefore, have been labeled as liquid ordered phase. While diffraction can be sensitive to small fluctuating membrane structures^16^,^17^, the structures observed here likely show a slower dynamics. It can be speculated that the presence of a solid support possibly leads to changes in the raft dynamics of the RBC membranes on a chip, as compared to the membrane in intact red blood cells.

The structural parameters of the three different membrane patches in Table 1 are in excellent agreement with values reported from model membrane studies. The two lipid domains were interpreted as manifestations of liquid ordered and liquid disordered phases. The *l*_*o*_ domains were found to be thicker (*d*_*HH*_ = 46 Å), with a relatively small area per lipid tail of *A*_*T*_ = 19 Å^2^, while the *l*_*d*_ domains are significantly thinner (41 Å) with a greater lipid tail area (*A*_*T*_ = 25 Å^2^), typical for a fluid structure. The average thickness of the peptide domains of 40.6 Å is compatible with the thickness of the membranes and support the assignment to integral peptides. The corresponding patch sizes are small, between about 20 and 30 Å.

While RBC membranes were previously reported to consist of ~52% proteins and ~40% lipids (including cholesterol)^4^, we observe a higher fraction of lipids (and cholesterol) and fewer peptides. X-ray diffraction is not sensitive to monomeric integral or peripheral peptides, but instead to larger integral peptide regions (the packing of peptide helices in the membrane core). These helical regions are likely part of larger trans-membrane proteins. Chemical analysis techniques or mass spectrometry may, therefore, result in a higher total concentration of peptides. The value determined in this work indicates that about 50% of the peptides in RBC membranes can be considered as integral membrane proteins.

There is growing evidence for an influence of various pharmaceuticals on lipid membrane organization and stability^67^. In particular, non-steroidal anti-inflammatory drugs (NSAID’s) have been shown to disturb bilayer structures in native and model membranes^68^,^69^. Aspirin is the most common NSAID and is known to interact with membranes^15^,^68^. Aspirin strongly perturbs model membrane structure in a concentration dependent manner and also influences human erythrocyte shape^70^. It decreases the hydrophobic surface barrier in mucosal membranes, leading to a diffusion of acid and gastrointestinal injury^71^ and impacts on protein sorting^72^. Aspirin was previously reported to partition in lipid bilayers and position itself in the lipid head group region^15^,^36^,^37^. Recently, an interaction between aspirin and cholesterol was reported, as aspirin was observed to reduce the volume of cholesterol plaques in model membranes at elevated cholesterol concentrations of ~40 mol%^36^. Aspirin was also reported to inhibit the formation of cholesterol rafts in fluid lipid membranes at physiological cholesterol concentrations^36^,^37^.

The effect of ASA on the molecular organization of RBC was determined by X-ray diffraction. Our main findings are that aspirin partitions in RBC membranes, and is located in the membrane head group region. Aspirin was found to reduce membrane thickness and increases lipid tail distances, indicative of a fluidification of the RBC membranes. This observation is in excellent agreement with the findings in model membranes. A cartoon of the structure of RBC membranes in the presence of ASA is shown in Fig. 5(c) and (d).

An interesting observation is that aspirin was preferably found in the *l*_*o*_ lipid regions of the RBC membranes. By calculating electron densities for the *l*_*d*_ and *l*_*o*_ domains in Fig. 4(c) and (d), a significant difference was observed for the *l*_*o*_ domains, only. This led to a thinning of the *l*_*o*_ domains and an increase in area per lipid tail, indicative of a fluidification of these more densely organized regions of the RBC membranes. This is in excellent agreement with observation in model membranes, where a strong interaction between model membranes in liquid ordered phases and aspirin was reported^15^,^36^,^37^. Also in model membranes, ASA was found to locate in the head group region of the bilayers and make bilayers more fluid and dissolve cholesterol structures, such as plaques and rafts.

ASA has a pronounced effect on the RBC membranes in our study: by reducing the thickness of the *l*_*o*_ regions it leads to a more uniform and overall more fluid membrane structure, where the *l*_*d*_ and *l*_*o*_ domains now have identical membrane thicknesses. This finding suggests aspirin exerts an effect on the physical properties of red-blood cell membranes, which may in turn help to understand the side-effects of aspirin and the low-dose-aspirin therapy. These RBC’s on a chip present a novel platform to test the interaction of other drugs and bacteria with RBC membranes and determine their molecular mode-of-action in the future.

## Conclusion

We prepared human red blood cell membranes on a chip, *i.e.* highly aligned multi-lamellar stacks of RBC membranes applied on silicon wafers. These solid supported RBC membranes are ideally suited for analysis using biophysical techniques. Based on the protocol for the preparation of red blood cell ghosts, small RBC vesicles were produced and applied onto functionalized silicon chips and annealed into multi-lamellar, planar membranes. Molecular structure of the RBC membranes was analyzed by high resolution X-ray diffraction.

The X-ray diffraction measurements present direct experimental evidence that RBC membranes consist of nanometer sized *l*_*o*_ and *l*_*d*_ lipid domains, as well as integral *α*-helical coiled-coil peptide domains. The composition of RBC’s was determined to be 30:45:25 (*l*_*o*_:*l*_*d*_:coiled peptides), indicating that about 50% of the proteins in RBC membranes are integral membrane proteins.

RBC membranes that contain up to 3 mM of ASA were prepared. We present experimental evidence that aspirin partitions in RBC membranes and preferably locates in the head groups region of the *l*_*o*_ lipid domains. ASA led to an increase of the lipid-lipid distance and a decrease of the membranes thickness, indicative of a fluidification of the RBC membranes.

## Materials and Methods

This research was approved by the Hamilton Integrated Research Ethics Board (HIREB) under approval number 1354-T. Informed consent was obtained from all blood donors. The authors confirm that all methods were performed in accordance with the relevant guidelines and regulations.

### Preparation of ghosts

The preparation of RBC ghosts was first published in 1963 by Dodge, Mitchell and Hanahan^1^: 10 mL of venous blood were drawn from a participating individual. The blood was collected in venous blood collection tubes from BD (Product Number: BD 367874), coated with sodium heparin as anticoagulant. The tube was centrifuged at 3,000 g for 10 min at room temperature. After this process, a clear separation between an erythrocyte fraction and a plasma fraction was observed. The white blood cells and platelets form a layer between those two fractions. In the original protocol, the RBC fraction was then filtered by a procedure by Beutler, West and Blume^73^, where the RBC fraction is pushed through a cellulose filter (details can be found in the Supplementary Material). This process was suggested to produce pure erythrocyte preparations without the remaining leucocytes and platelets. While this protocol is well established and widely used in blood cell investigations (see, for instance^74^, for a recent review), the ghost solution produced by this protocol did not result in well developed multi-lamellar membrane stacks when applied on silicon wafers. Cellulose particles were observed under the microscope in the solution after passing through the filter (shown in the Supplementary Material in Fig. S1), which likely inhibit the formation of well-ordered membrane stacks.

In order to avoid contamination with cellulose, the RBC solution was purified through centrifugation using the following protocol: The supernatant in the separated blood sample was removed using a pipette. PBS was added to the precipitate to achieve a volume of 10 mL and centrifuged at 3,000 g for 10 min. This process was repeated twice.

50 *μ*L of the RBC solution was then mixed with 1 mL of buffer solution in a 1.5 mL reaction tube. For the buffer, 16 mL of PBS and 484 mL of 18.2 MΩ ⋅ cm ultra pure water were mixed and stored at 0 °C. The solution was buffered with potassium hydroxide and hydrochloric acid to a pH of 8. This creates a hypotonic solution for the RBCs, resulting in an influx of water into the cells and their lysis. The diluted solution is vortexed for 10 s to prevent clumping. After vortexing, the reaction tube is immediately placed in ice for 30 min to slow down the re-closing of the burst cells.

**Figure 6:**
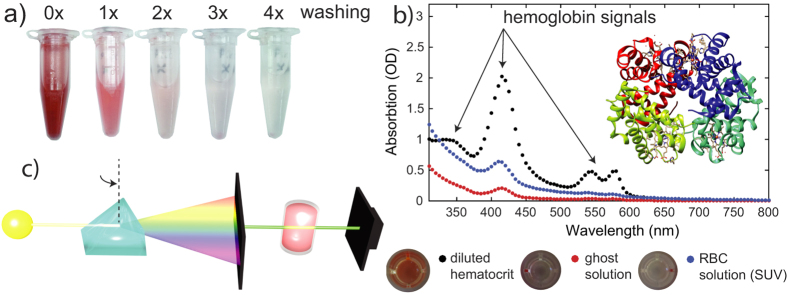
Removal of hemoglobin from the erythrocyte blood fraction after induced lysis in hypotonic buffer. (**a**) Ghost samples lose their characteristic red color through sequential centrifugation and washes. (**b**) Comparison of UV-vis absorbance curves at different stages within ghost preparation. Characteristic hemoglobin absorbance signatures are significantly reduced in the final solution after the procedure. (**c**) Schematic of the UV-vis setup.

Samples were then centrifuged at 18,000 g for 30 min at 0 °C. After the centrifugation, a pellet is formed at the bottom of the reaction tube. The supernatant was removed by pouring the reaction tube in a beaker. 1 mL buffer solution was added to the pellet and the solution was votexed for 10 s and centrifuged for 15 min at 18,000 g and 0 °C. This process of centrifugation and removal of the supernatant was repeated 4 times. During this washing, most of the hemoglobin is removed, resulting in a transparent, colorless solution. Figure 6(a) shows images of the reaction tube after different numbers of washing steps.

The removal of hemoglobin was quantitatively checked using ultraviolet-visible spectroscopy (UV-vis). The corresponding data is shown in Fig. 6(b). The characteristic hemoglobin absorption bands at 335 nm, 416.4 nm 543 nm and 577 nm decrease in every step; the hemoglobin content of the final solution was found to contain less than 2% of the original content.

By weighing the pellets after each step of the preparation, this procedure results in solutions with typical mass concentration of RBC’s of ~0.3 mg/mL. To increase the concentration, pellets from 24 such reaction tubes were collected and centrifuged at 18,000 g for 15 min. The supernatant was removed and the tube was refilled with buffer solution to the 1 mL mark of the tube. This results in a solution with a final mass concentration of ~7 mg/mL.

**Figure 7:**
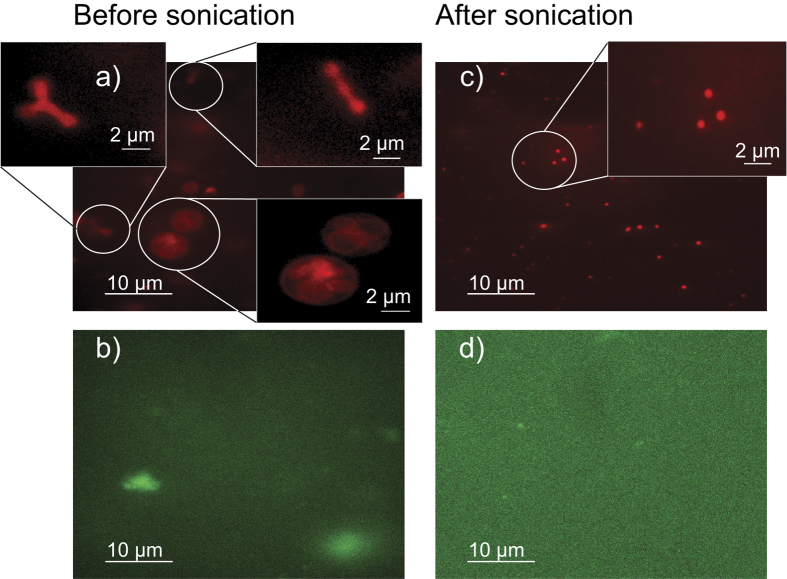
Fluorescence microscopy images of the ghost solution before and after sonication. The membrane was labelled using DiI in parts (**a**) and (**c**), while Alexa Fluor 488 labelled phalloidin was used to label the F-actin network in (**b**) and (**d**). Before sonication, ghosts of highly irregular shape and a large size distribution are observed including ‘ghosts inside of ghosts’. The solution also contains large clusters of actin. Small vesicles with a uniform size distribution are observed after sonication and no actin particles (within the resolution of the microscope used).

The ghost solution was analyzed by fluorescence microscopy, as shown in Fig. 7(a) and (b). The red blood cell membrane was fluorescently labeled in part (a) using 1,1′-Dioctadecyl-3,3,3′,3′-Tetramethylindocarbocyanine Perchlorate (DiI). The image shows a mix of multi-lamellar and uni-lamellar ghosts with a highly irregular shape and a large distribution of shapes and sizes, from round to long, more chain-like objects including vesicles that contain several smaller vesicles. These shapes are likely related to the presence of a cytoskeleton, whose main components are spectrin and actin in RBC^75^. To analyze this network, Alexa Fluor 488 labelled phalloidin was used to label the F-actin network in Fig. 7(b). Structures of ~5 *μ*m were observed, indicative of the presence of actin.

As indicated below, the variation in size and shape of the ghosts, and the presence of an actin network likely prevents the formation of well defined, solid supported multi-lamellar RBC. To achieve a more uniform distribution of vesicle sizes and shapes, the RBC solution was tip sonicated 10 times for 5 s, each, in order to form small vesicles with a uniform size distribution. The result of the sonication process is shown in Fig. 7(c) and (d). In part (c), the membrane was fluorescently labeled using DiI. Small dots were observed, indicative of small vesicles of ~50 nm, beyond the resolution limit of the microscope.

After sonication, no more particles were observed within the resolution of the microscope used. In order to separate the SUVs and remaining actin, the solution was centrifuged for 30 min at 20,000 g. Since SUVs can only sediment in ultracentrifuges at 120,000 g when centrifuged for more than 30 min^76^, the pellet contains actin polymers and potential larger and multi-lamellar vesicles, while the SUVs stay in the supernatant. This supernatant was found to be ideal for the formation of solid supported, multi-lamellar RBC membranes, as will be discussed below.

### Silicon wafer preparation

All membranes were prepared on single-side polished silicon wafers. 100 mm diameter, 300 *μ*m thick silicon wafers were pre-cut into 10 × 10 mm^2^ chips. The wafers were functionalized for deposition of the ghost solution by either preparing a hydrophobic or hydrophilic surface.

To create a hydrophobic silicon surface, the wafers were pre-treated by sonication in dichloromethane (DCM) at 40 °C for 25 min. This treatment removes all organic contamination and leaves the surface in a hydrophobic state. Each wafer was then thoroughly rinsed three times by alternating with ~50 mL of ultra pure water with a resistivity of 18.2 MΩ ⋅ cm and HPLC grade methanol.

To create a hydrophilic state, the wafers were cleaned by immersion in an 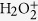 sulfuric acid mixture (volume fraction of 70% concentrated H_2_SO_4_, 30% H_2_O_2_ at 40 °C, Piranha solution) for 30 min on a 3D orbital shaker (VWR) set to tilt angle 1 and speed 15). This strongly oxidizing combination removes all organic contaminants on the surface, but does not disturb the native silicon oxide layer. Each wafer was then thoroughly rinsed with ~50 mL of ultra pure water with a resistivity of 18.2 MΩ ⋅ cm.

### Fabrication of highly oriented, multi-lamellar solid supported RBC membranes

**Figure 8:**
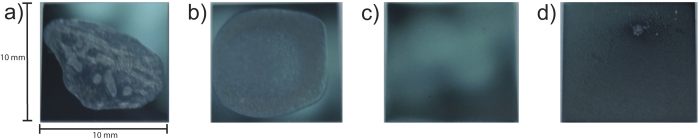
Photos of the silicon chips after (**a**) application of the RBC solution on a hydrophilic wafer and fast drying, (**b**) application on a hydrophobic wafer after slow drying. (**c**) and (**d**) show hydrophilic wafers after slow drying and slow drying and annealing, respectively. See text for details.

The ghost solution did not spread well on hydrophobic silicon wafers, as shown in Fig. 8(a). For this wafer, 100 *μ*L of concentrated ghosts solution was applied onto a 10 × 10 mm^2^ hydrophobic silicon wafer mounted on a leveled hot plate at a temperature of 40 °C. The solution was applied slowly using a 100 *μ*L syringe to avoid spill, and the wafer typically dried within ~10 min. The membrane film was found not to cover the entire wafer and showed several wrinkles.

Slowly drying the solution to allow more time for the solution to spread and membranes to form was achieved by placing the wafers in a leveled desiccator for 5 days at 97.6 ± 0.5% relative humidity using a saturated K_2_SO_4_ salt solution. The slow drying resulted in a smoother film, however, still incomplete coverage of the substrate, as shown in Fig. 8(b).

Figure 8(c) shows a hydrophilic wafer prepared by applying 100 *μ*L of concentrated SUV solution and dried for 5 days at 97.6 ± 0.5% relative humidity. The solution covered the entire wafer indicating a homogeneous mass distribution. We could, however, only detect weak signals of membrane stacking in this sample and picture the morphology of the membranes as depicted in Fig. 1(d), as small vesicles that have been dried out on the silicon substrate. This situation is similar to the preparation of single solid supported bilayers through vesicle fusion^77^,^78^, where small bilayer patches initially develop on the substrate, and eventually undergo a transition into a large uniform single bilayer^77^. Substrates are typically annealed for 72 h at 55 °C in an oven in air. The energy barrier for forming a lamellar structure can be overcome through gentle heating and the lamellar membrane organization becomes energetically more favorable, as it minimize the bending energy.

However, using the same procedure and heating the RBC membranes in an oven led to destruction of the membrane film. The silicon chips were, therefore, incubated at different temperatures and under relative humidities between 50% and 100% RH by placing them in a closed container and exposure to different saturated salt solutions. The best results were obtained when the RBC chip was incubated at 50 °C and 95.8 ± 0.5% relative humidity in a saturated K_2_SO_4_ salt solution for 5 days, which resulted in the photo in Fig. 8(d). In this protocol, annealing of the RBC membranes at high temperature and humidity leads to the formation of lamellar membrane structures through membrane fusion.

The number of stacked RBC membranes on one of these chips can be estimated as follows: 100 *μ*L of the 7 mg/mL RBC contain ~2 ⋅ 10^−6^ mol (when assuming an average molecular weight of the membranes of 400 g/mol). By using the values in Table 1, areas of *l*_*o*_ and *l*_*d*_ lipids can be estimated to 38 and 50 Å^2^, and 95 Å^2^ (2*π* ⋅ (11/2)^2^) for the peptides. By using the volume fractions of the different components, the average area per particle is then calculated to 0.3 ⋅ 38 Å^2^ (*l*_*o*_) + 0.45 ⋅ 50 Å^2^ (*l*_*d*_) + 0.25 ⋅ 95 Å^2^ (peptides) ≈ 58 Å^2^. The total area of the RBC membranes is calculated to (2 ⋅ 10^−6^) ⋅ (6 ⋅ 10^23^) ⋅ (58 ⋅ 10^−20^) m^2^ ≈ 0.7 m^2^. Taking into account the area of a silicon chip of 1 ⋅ 10^−4^ m^2^, this results in about 700 stacked RBC membranes per chip.

### Preparation of RBC/aspirin complexes

In order to prepare complexes of RBC membranes containing increasing amounts of aspirin, a solution of 9 mg/mL acetylsalicylic acid (molecular weight 180 g/mol) in 18.2 MΩ ⋅ cm water was prepared. 2 *μ*L, 3 *μ*L, 4 *μ*L, 5 *μ*L, and 6 *μ*L of this solution were added to 100 *μ*L of the final RBC solution resulting in acetylsalicylic acid concentrations of 1 mM, 1.5 mM, 2 mM, 2.5 mM and 3 mM. The resulting solutions were applied onto silicon wafers and dried slowly and incubated for 5 days following the above protocol. We note that in this study the aspirin was added to the RBC solution before it was applied onto the silicon wafer. This protocol ensures perfect mixing of RBC membranes and aspirin and makes sure that the aspirin is distributed uniformly throughout the membrane stack.

The molar concentration of ASA in the RBC membranes can be estimated as follows: between 2 and 5 *μ*L of the 9 mg/mL ASA solution were added to the membrane solution, resulting in between 1 ⋅ 10^−7^ and 2.5 ⋅ 10^−7^ mol. 100 *μ*L of the 7 mg/mL RBC contain ~2 ⋅ 10^−6^ mol (when assuming an average molecular weight of the membranes of 400 g/mol). This results in molar ASA concentrations between 5–10 mol%, *i.e.*, 1 ASA molecule per 10 to 20 lipid molecules. This ASA concentration is elevated as compared to plasma concentrations of typically less than 1 mol%, however, comparable to ASA concentrations typically used in the literature^79^.

#### Optical Microscopy and total internal reflection fluorescence microscopy (TIRF)

AlexaFluor 488-phalloidin (Invitrogen, Life Technologies, Burlington, ON) was utilized to stain the actin filaments within the cells so that these structures may be visualized with the fluorescence microscope. To stain actin filaments, the RBC membranes were first permeabilized using a 0.2% Triton-X 100 solution in ultra-pure water with an incubation of 5 minutes. The substrates were then washed with ultra-pure water, and 5 *μ*L of stock phalloidin in 200 *μ*L of ultra-pure water was added to each sample and incubated for 20 minutes at room temperature. Then, the staining solution was replaced with ultra-pure water. In order to avoid a shrinking of the ghost vesicles because of the osmotic pressure, ultra-pure water was used instead of PBS, as proposed in the original protocol. 1,1′-Dioctadecyl-3,3,3′,3′-Tetramethylindocarbocyanine Perchlorate (Sigma-Aldrich) was used to label the membranes.

Images were acquired through a LEICA DMI6000 B inverted microscope equipped with a Spectral Laser Merge Module for multi-wavelength illumination (Spectral, Richmond Hill, ON), adaptive focus control, a motorized X-Y stage (MCL Micro-Drive, Mad City Labs Inc., Madison, WI), a piezo X-Y-Z stage (MCL Nano-Drive, Mad City Labs Inc., Madison, WI), a LEICA 100x/1.47NA oil-immersed TIRF objective and an Andor iXon Ultra EMCCD camera. Excitation was provided by 488 and 647 nm diode-pumped solid-state lasers with 40 mW and 60 mW output power respectively (Spectral, Richmond Hill, ON). The samples were pipetted into glass-bottom 35 mm Petri dishes (MatTek Co., Ashland, MA), and visualized using the inverted microscope with illumination in widefield fluorescence mode.

#### UV-vis spectroscopy

Ultravioletvisible spectroscopy (UV-vis) was obtained using a M1000Pro Plate reader from Tecan. The technique is depicted in Fig. 6(c): the absorption of light in the visible and adjacent (near-UV and near-infrared) ranges is detected. Hemoglobin shows characteristic absorption lines at 335 ± 0.4 nm, 416.4 ± 0.2 nm 543 ± 0.8 nm and 577 ± 0.4 nm^80^. In order to prepare a sufficiently diluted RBC solution, 50 *μ*L of the erythrocytes fraction was mixed with 1 mL PBS. 400 *μ*L of this solution was afterwards diluted with 400 *μ*L PBS. This dilution procedure has been repeated three times. For the measurement, a 96-plate from Costar was used. 200 *μ*L of the diluted blood solution, the ghosts solution and the RBC solution were filled in the chambers of the plate. The absorption spectrum for each sample was scanned for wavelengths between 310 nm and 800 nm.

#### X-Ray Diffraction

X-ray scattering data was obtained using the Biological Large Angle Diffraction Experiment (BLADE) in the Laboratory for Membrane and Protein Dynamics at McMaster University. BLADE uses a 9 kW (45 kV, 200 mA) CuK*α* rotating anode at a wavelength of 1.5418 Å. Both source and detector are mounted on movable arms such that the membranes stay horizontal during the measurements. Focusing multi-layer optics provides a high intensity parallel beam with monochromatic X-ray intensities up to 10^10^ counts/(mm^2^ ⋅ s). This beam geometry provides optimal illumination of the solid supported membrane samples to maximize the scattering signal. A sketch of the scattering geometry is shown in Fig. 2(a). Note that there is no risk of sample damage using this in-house technique because of the large beam size and relatively low intensity of the X-ray beam as compared to synchrotron sources.

The result of an X-ray experiment is a 2-dimensional intensity map of a large area of the reciprocal space, as sketched in Fig. 2. The corresponding real-space length scales are determined by *d* = 2*π*/|*Q*| and cover length scales from about 2.5 to 100 Å. All scans were measured at 28 °C and 50% relative humidity (RH) hydration. As depicted in Fig. 2(a), the wafers were oriented in the X-ray diffractometer, such that the *q*_||_-axis probed lateral structure, parallel to the wafer surface, and the perpendicular axis, *q*_*z*_, probed out-of-plane structure, perpendicular to the substrate.

The experimental errors were determined as follows: Errors for peak positions, peak width and peak height are determined as the fit standard errors, corresponding to 95% confidence bounds, equivalent to 2 standard deviations, *σ*. Errors for calculated parameters, such as peak area, were then calculated by applying the proper error propagation.

#### Calculation of Electron Densities

The out-of-plane structure of the membrane was determined using specular reflectivity. The relative electron density, *ρ*(z), is approximated by a 1-dimensional Fourier analysis^15^,^81^:


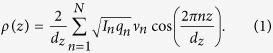


*N* is the highest order of the Bragg peaks observed in the experiment. The integrated peak intensities, *I*_*n*_, are multiplied by *q*_*n*_ to receive the form factors, *F*(*q*_*n*_)^15^,^81^. The bilayer form factor *F*(*q*_*z*_), which is in general a complex quantity, is real-valued in the case of centro-symmetry. The phase problem of crystallography, therefore, simplifies to the sign problem *F*(*q*_*z*_) =  ± |*F*(*q*_*z*_)| and the phases, *v*_*n*_, can only take the values ± 1. The phases *v*_*n*_ are needed to reconstruct the electron density profile from the scattering data following Eq. (1). When the membrane form factor *F*(*q*_*z*_) is measured at several *q*_*z*_ values, a continuous function, *T*(*q*_*z*_), which is proportional to *F*(*q*_*z*_), can be fitted to the data^15^,^81^.


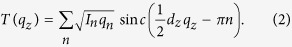


Once an analytical expression for *T*(*q*_*z*_) has been determined from fitting the experimental peak intensities, the phases *v*_*n*_ can be assessed from *T*(*q*_*z*_). The phase array *v*_*n*_ = [−1 −1 1 −1 1] was used for all samples. A sample *T*(*q*_*z*_) is shown in Fig. S3 in the Supplementary Material.

The electron densities determined by Eq. (1) are on a relative scale. In order to compare the electron densities in Figs 3(c) and 4(c), *ρ* in the membrane center at *z* = 0 was set to 0 and the electron density at the boundaries (*z* values between 25 and 30 Å depending on the lamellar spacing), which probe the water layer between the stacked membranes, were scaled to the electron density of water of *ρ* = 0.33 e^−^/Å^3^.

#### Membrane orientation

To determine the degree of orientation of the membranes in the stack the correlation peak intensities were integrated as function of the meridonal angle *φ* (the angle relative to the *q*_*z*_ axis). The corresponding intensity was fit with a Gaussian distribution centered at 0, which was then used to calculate the degree of orientation using Hermans orientation function:


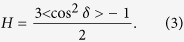


The degree of orientation, *H*, of the RBC membranes was measured to be 90.9%.

#### Determination of domain sizes

The average size of the different lipid and peptide domains was estimated from the widths of the corresponding in-plane correlation peaks in Fig. 2(d) by applying Scherrer’s equation^82^:


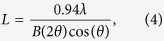


where *λ* is the wavelength of the X-ray beam, *θ* is the diffraction angle and *B*(2*θ*) is the width of the correlation peak in radians. Scherrer’s equation is an established way to estimate crystallite sizes of up to ~100 nm in X-ray diffraction experiments. We note that the equation has limitations to quantitatively determine sizes of small domains of a few nanometers, only. The values that we determine, therefore, present upper limits of the domain sizes.

## Additional Information

**How to cite this article**: Himbert, S. *et al*. The Molecular Structure of Human Red Blood Cell Membranes from Highly Oriented, Solid Supported Multi-Lamellar Membranes. *Sci. Rep.* **7**, 39661; doi: 10.1038/srep39661 (2017).

**Publisher's note:** Springer Nature remains neutral with regard to jurisdictional claims in published maps and institutional affiliations.

## Supplementary Material

Supplementary Information

## Figures and Tables

**Table 1:** The lamellar spacings, molecular distances, *a*, and tail areas, *A*_*T*_, were determined from the peak positions in the out-of-plane and in-plane scattering, respectively.

	*l*_*o*_ lipid domains	*l*_*d*_ lipid domains	peptide domains
*d*_*z*_	59.2 ± 0.5 Å	51.6 ± 0.02 Å	40.6 ± 0.06 Å
*d*_*HH*_	46.0 ± 0.5 Å	41.0 ± 0.02 Å	—
*d*_*w*_	13.2 ± 0.5 Å	10.6 ± 0.02 Å	—
*a*	4.69 ± 0.27 Å	5.39 ± 0.03 Å	10.88 ± 0.22 Å
*A*_*T*_	19.04 ± 1.10 Å	25.18 ± 0.13 Å	—
*ξ*	16 ± 3 Å	29 ± 2 Å	28 ± 3 Å

Tail areas include the area of cholesterol molecules. Membrane thickness, *d*_*HH*_, and the thickness of the water layer, *d*_*w*_, were determined from the electron densities *ρ*(*z*). Domain sizes, *ξ*, were determined from the width of the in-plane correlation peaks. Experimental errors are given.
