# Supplementary material for: The Molecular Structure of Human Red Blood Cell Membranes from Highly Oriented, Solid Supported Multi-Lamellar Membranes
Source: Sci Rep. 2017 Jan 3;7:39661. doi: 10.1038/srep39661 (PMC5206716; doi:10.1038/srep39661)
Supplement: Supplementary Information [file srep39661-s1.pdf]

Supplementary Information to: The Molecular Structure of Human Red Blood Cell Membranes from Highly Oriented, Solid Supported Multi-Lamellar Membranes

Sebastian Himbert, Richard J. Alsop, Markus Rose, Laura Hertz, Alexander Dhaliwal, Jose M. Moran-Mirabal, Chris P. Verschoor, Dawn M. E. Bowdish, Lars Kaestner, Christian Wagner, and Maikel C. Rheinstädter*

(Dated: November 11, 2016)

* Department of Physics and Astronomy, McMaster University, ABB-241, 1280 Main Street West, Hamilton, Ontario L8S 4M1, Canada; Phone: +1-(905)-525-9140-23134, Fax: +1-(905)-546-1252, E-mail:rheinstadter@mcmaster.ca

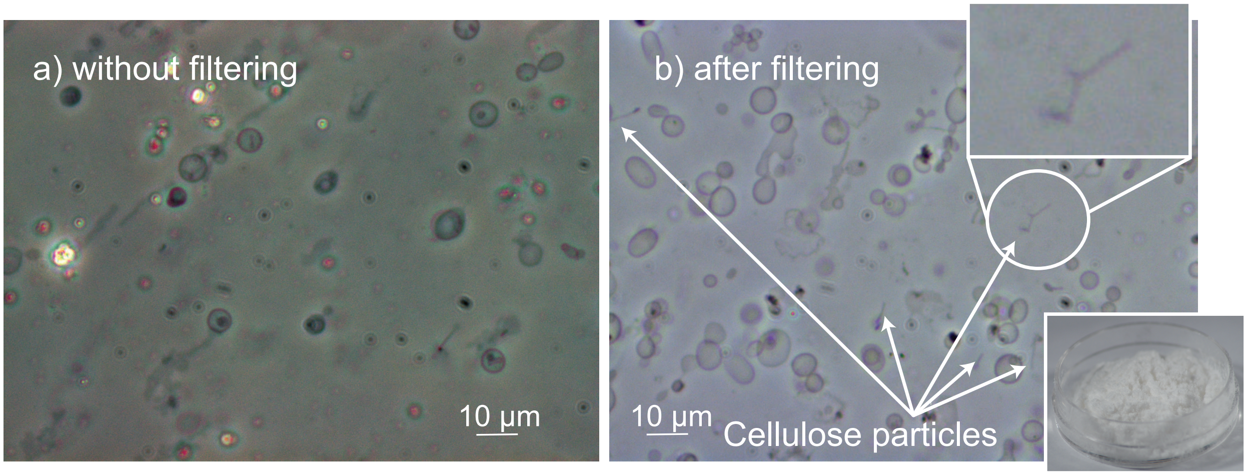

Supplementary Information, Figure S1. Optical microscope images of the ghost solution with (a) and without cellulose filtering (b), as described in the text. In both images, ghosts of varying size and shape are observed. Cellulose particles are observed in addition in the filtered solution.

We initially followed the standard filtering procedure described by Beutler, West and Blume [1]: 1.8 g of α-cellulose and σ-cellulose were added to 100 mL of 0.9% phosphate buffered saline (PBS, Sigma Aldrich). 10 mL of this solution was filled in a syringe furnished with a filter paper at the bottom [1] and pushed through the filter, such that the cellulose aggregates at the bottom form a sponge-like filter. A mixture of 9 mL PBS and 1 mL plasma was then filled into the syringe an let percolate through the filter as well. Finally, 1 mL of a 1:1 mixture of the erythrocytes fraction and PBS were pushed through the filter and the filtrate was disposed. This process was suggested to isolate erythrocytes from leucocytes and platelets. While leucocytes and platelets remain in the filter, the erythrocytes can pass the filter. To support this process, the filter is washed with 10 mL PBS and the filtrate was collected and used for the lysing process.

Microscope images of the ghost solution before and after the filtering process are shown in Fig. S1. Before filtering (Fig. S1 a), round vesicles and fused, chainlike vesicles of μm size are observed. After the filtering process in Fig. S1 b), additional polymeric objects were observed under the microscope, likely related to the presence of cellulose particles in the solution after passing through the filter.

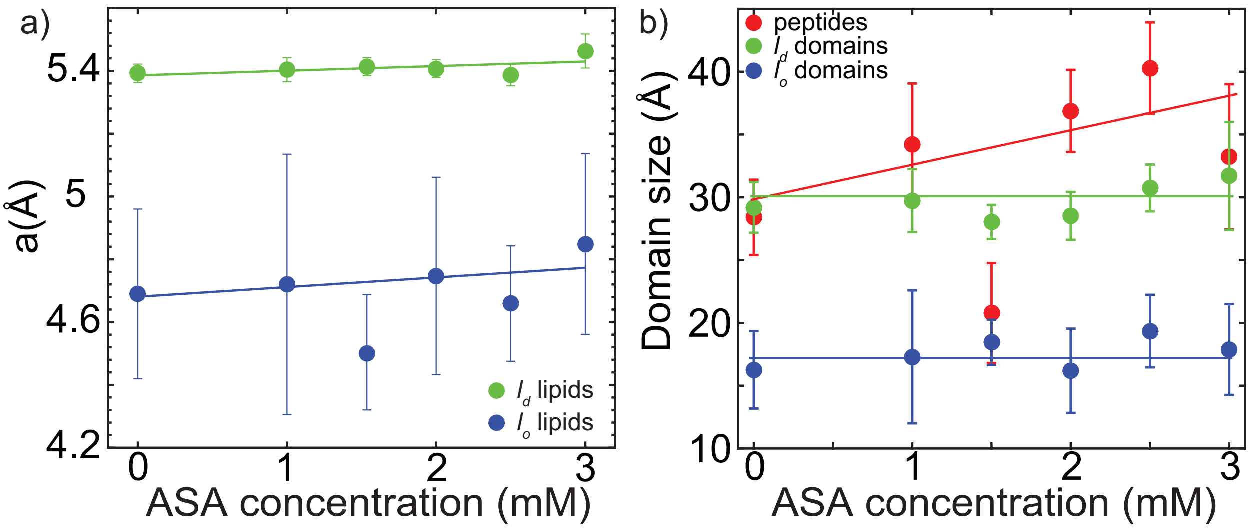

Supplementary Information, Figure S2. a) The positions of the lipid correlation peaks move to smaller $q_{||}$-values indicative of an increase of the lipid tail distances. The spacing, a, in the l_o domains slightly increases as compared to the l_d domains, indicating that ASA has a stronger affinity and impact on l_o lipids. b) While the size of lipid l_o and l_d domains is basically not affected by increasing the ASA concentration (within the experimental errors of this experiment), the size of the peptide domain slightly increased from ∼30 Å to ∼38 Å.

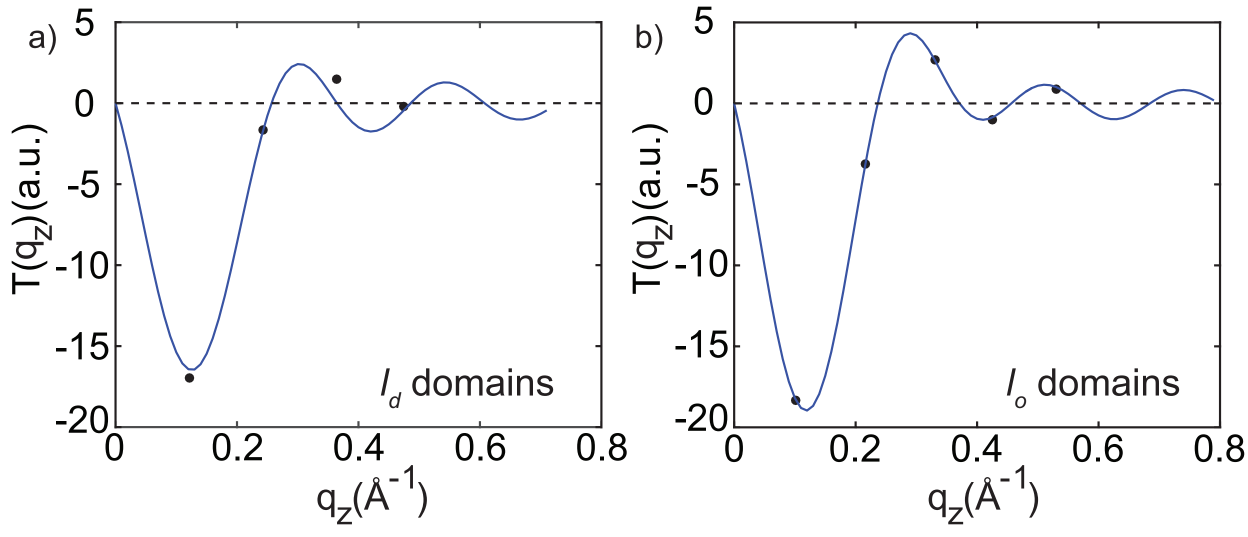

Supplementary Information, Figure S3. Sample $T(q_z)$ following Eq. (2) for the l_d (a) and the l_o (b) lipid signal, which was used to assess the phases required for the Fourier transformation of the electron densities. $T(q_z)$ is proportional to the membrane form factor.

[1] Beutler E, West C, Blume K. The removal of leukocytes and platelets from whole blood. J Lab Clin Med. 1976;88(2):328–333.
